# High Psychological Impact of Covid‐19 on French Healthcare Workers: An Observational Cohort Study of PTSD, Depression and Burn‐Out

**DOI:** 10.1111/hex.70401

**Published:** 2025-08-21

**Authors:** Wissam El‐Hage, Alexandre Lemé, Myriam Blanchin, Eric Bui, Hala Kerbage, Sarah Ibouhsissen, Aude Allemang‐Trivalle, Valérie Gissot, Bénédicte Gohier, Coraline Hingray, Philippe Birmes, Eric Fakra, Nathalie Prieto, Cédric Lemogne, Marie‐Odile Krebs, Bruno Aouizerate, Isabelle Jalenques, Pierre Vidailhet, Anne Sauvaget, Agnès Caille

**Affiliations:** ^1^ Centre Régional du Psychotraumatisme CVL, CHRU de Tours Tours France; ^2^ UMR 1253, iBraiN, Université de Tours, Inserm Tours France; ^3^ CIC1415, CHRU de Tours, Inserm Tours France; ^4^ SPHERE U1246, Université de Tours, Université de Nantes, Inserm Tours France; ^5^ Université de Caen Normandie et CHU Caen Caen France; ^6^ Department of Child and Adolescent Psychiatry Saint Eloi University Hospital Montpellier France; ^7^ Center for Epidemiology and Population Health (CESP), INSERM U1018, Developmental Psychiatry Paris‐Saclay University Paris France; ^8^ Université Angers, LPPL, SFR CONFLUENCES, Service de Psychiatrie, CHU Angers Angers France; ^9^ Pôle Hospitalo‐Universitaire de Psychiatrie d'Adultes du Grand Nancy, Centre Psychothérapique de Nancy Laxou France; ^10^ Toulouse NeuroImaging Centre, University of Toulouse, Inserm, UPS Toulouse France; ^11^ Pôle Universitaire de Psychiatrie, CHU Saint‐Etienne Saint‐Etienne France; ^12^ Cellule d'Urgence Médico‐Psychologique, Centre Régional du Psychotraumatisme, Hôpital Edouard Herriot Lyon France; ^13^ Université Paris Cité and Université Sorbonne Paris Nord, Inserm, INRAE, Center for Research in Epidemiology and StatisticS (CRESS) Paris France; ^14^ Service de Psychiatrie de l'adulte, AP‐HP, Hôpital Hôtel‐Dieu Paris France; ^15^ Laboratoire de Physiopathologie des maladies Psychiatriques, UMR S1266, Institut de Psychiatrie et Neurosciences de Paris, Université Paris Descartes, Inserm Paris France; ^16^ Centre de Référence Régional des Pathologies Anxieuses et de la Dépression, Pôle de Psychiatrie Générale et Universitaire, CH Charles Perrens Bordeaux France; ^17^ Laboratoire Nutrition et Neurobiologie intégrée, UMR INRAE 1286 Université de Bordeaux Bordeaux France; ^18^ Clermont Auvergne Université, CNRS, CHU Clermont‐Ferrand, Service de Psychiatrie de l'Adulte A et Psychologie Médicale, Clermont Auvergne INP, Institut Pascal Clermont‐Ferrand France; ^19^ Pôle de Psychiatrie, Santé Mentale et Addictologie, Hôpitaux Universitaires de Strasbourg; Fédération de Médecine Translationnelle de Strasbourg Université de Strasbourg Strasbourg France; ^20^ Nantes Université, CHU Nantes, Movement ‐ Interactions ‐ Performance, MIP Nantes France

**Keywords:** burn‐out, Covid‐19, depression, healthcare workers, PTSD

## Abstract

**Introduction:**

The Covid‐19 outbreak hit the world forcing public institutions to rethink their core functioning. Healthcare workers (HCWs) were particularly at risk for negative mental health outcomes, given their direct exposure to many pandemic‐related stressors. Our study aimed to assess the psychological outcomes of French HCWs during the Covid‐19 pandemic, including PTSD, depression and burn‐out.

**Methods:**

This study presents the baseline data of a large cohort study conducted during the pandemic. Participants were 849 French HCWs (mostly single women, working as nurses) assessed for PTSD with the PCL‐5 (PTSD Checklist for DSM‐5), for depression with the PHQ‐9 (Patient Health Questionnaire), and for burn‐out and compassion satisfaction with the ProQOL (Professional Quality of Life). Other pandemic‐related variables were also collected, including characteristics related to participants' Covid‐19 experience, as well as psychological and clinical measures.

**Results:**

The prevalence rates of PTSD, moderately severe depression, and high risk for burn‐out were 17.7% (*n* = 146), 14.0% (*n* = 118) and 0.9% (*n* = 7), respectively. Further, findings suggested that working conditions were challenging, though they still found pride in their work. Univariate analyses showed that being a woman, not being a physician, and lacking protective equipment were each associated with higher scores on several negative psychological outcomes (PTSD, depression, burn‐out and secondary traumatic stress), though not consistently across all dimensions. Notably, satisfaction scores on the ProQOL did not differ by gender and were higher among physicians. In the multivariate analyses, not being a physician was independently associated with higher levels of PTSD, depression and burn‐out symptoms. Being a woman was independently associated with increased PTSD symptom severity. Higher levels of positivity—defined as a general tendency to view life and experiences with an optimistic and constructive outlook, as measured by the Positivity Scale—were associated with lower symptom scores across all three psychological outcomes.

**Conclusion:**

This study highlights the considerable psychological toll of the Covid‐19 pandemic on French HCWs, particularly among nurses and women, and underscores the protective role of individual psychological resources such as positivity. As one of the largest cohort studies conducted during the pandemic in France, it provides critical evidence to guide public health strategies, institutional policies and mental health interventions. The findings emphasise the urgent need to strengthen occupational mental health support systems and to promote resilience‐building approaches among healthcare professionals facing prolonged crisis conditions.

## 1 Introduction

Covid‐19 was an unprecedented pandemic, with a sudden global outbreak caused by a novel coronavirus, SARS‐CoV‐2 [1]. The pandemic exposed the fragility of healthcare systems worldwide. The rapid spread of the disease and the swiftly rising death toll [2] compelled authorities across the globe to implement urgent and wide‐reaching measures. Every aspect of society was deeply affected and transformed, including the political, economic, cultural and, above all, healthcare systems. The crisis redefined our understanding of health as a global concern, with healthcare workers (HCWs) at the forefront of the response.

France, as one of the countries hardest hit during the early phase of the pandemic [3], implemented evolving public health policies throughout the various waves of the outbreak. These included both national and local measures, such as border closures, nighttime curfews, and restrictions on hospital visits, as well as responses to shortages in personal protective and testing equipment [4, 5]. While these measures mitigated some aspects of the outbreak, their effectiveness was variable. For example, quarantine measures alone were estimated to reduce virus transmission by 84% in 2020 [6].

Nonetheless, the pandemic's long‐lasting consequences continue to be observed, especially in terms of psychological outcomes. The passage of time has enabled research teams worldwide to examine these effects, highlighting increases in various mental health conditions in the general population, including acute stress disorder, post‐traumatic stress disorder (PTSD), depressive symptoms, and anxiety [7, 8].

Understanding the psychological impact of the pandemic on HCWs quickly emerged as a critical priority—both to assess the full burden of the pandemic and to inform targeted interventions aimed at preserving the healthcare workforce. Even before the pandemic, HCWs were recognised as a population vulnerable to psychological distress, due to frequent exposure to high‐stress environments and difficult working conditions [9, 10]. The pandemic exacerbated these challenges, with numerous studies reporting high rates of mental health problems such as depression, anxiety and sleep disorders among HCWs. Several risk factors have been identified, including being female or working on the frontline [11, 12, 13], as well as contextual factors such as access to protective equipment, psychological support and reliable information—all of which significantly influenced psychological outcomes [14, 15, 16]. A recent umbrella review and meta‐analysis of 87 meta‐analyses, encompassing 1,846 non‐overlapping studies and over 9.4 million HCWs, confirmed the high prevalence of psychological distress during the Covid‐19 pandemic [17]. Reported prevalence ratios ranged from 20% for PTSD to 44% for burn‐out, with intermediate values for depressive symptoms, anxiety, psychological distress, perceived stress, sleep problems and insomnia. Interestingly, follow‐up analyses revealed little variation in mental health outcomes across job categories or sex, suggesting that the psychological burden was widespread across the healthcare workforce. These findings are echoed by more focused studies, such as a large multicentre cross‐sectional study by Jing et al. [18], which reported alarming rates of mental health problems among HCWs, including occupational burn‐out (44%), depressive symptoms (70%), anxiety (47%) and PTSD (37%). This study identified both protective and risk factors: older age, female gender, higher income, more doses of the Covid‐19 vaccine, and higher levels of mindfulness, resilience and perceived social support were associated with lower burn‐out. Conversely, being a nurse, working in a Covid‐19 treatment unit, perceiving a high risk of infection and experiencing high work intensity were linked to greater psychological vulnerability.

It is important to note that the present study does not rely on a representative sample of the entire French HCWs population. Participants were recruited within the framework of the HARD (HealthcAre woRkers coviD‐19) study, which included a psychological intervention targeting individuals with significant psychological symptoms. As such, this cohort represents a particularly vulnerable subgroup of HCWs who were already experiencing psychological distress and actively seeking support. This context is essential to understanding the elevated prevalence of psychiatric symptoms and psychotropic medication use observed in the sample, and findings should be interpreted accordingly.

Our study aims to assess the prevalence and severity of major psychological outcomes in a French HCWs cohort and to explore the associated factors. Our specific objectives were to:

– Describe the socio‐demographic characteristics and Covid‐19 experience within that cohort;
– Estimate the prevalence of symptoms for three main psychological outcomes: PTSD, depression and burn‐out;
– Evaluate additional psychological outcomes, including post‐traumatic growth and self‐improvement, childhood trauma, anxiety symptoms, coping strategies to stressful events, positivity, health status, lived traumatic events, and suicidal ideation;
– Identify the variables associated with the presence and severity of these main and additional psychological outcomes.

The collected data could serve as a base for prevention programmes to better answer a potentially similar pandemic in the future.

## 2 Methods

### 2.1 Study Design

This study presents the findings from the larger HARD study [19], which was designed as a prospective observational cohort study incorporating a *Trial within a Cohort* design. The overall cohort was followed for 1 year, with assessments conducted at baseline and at months 3, 6, 9 and 12. Within this cohort, an interventional component was embedded: a pragmatic, randomised, open‐label, superiority trial evaluating the efficacy of a psychological intervention for participants presenting with significant psychological symptoms. The present manuscript focuses exclusively on the baseline data from the overall cohort. Results from the randomised intervention will be reported separately in a future publication.

### 2.2 Ethics

The study protocol received approval from the Sud‐Ouest et Outremer I Ethical Committee (No. 1‐20‐046 ID 48680). All enrolled participants provided an electronic informed consent, wherein they were briefed on the protocol and the study's objectives and granted permission for their data to be utilised in observational research.

### 2.3 Study Population

To conduct this study, we enrolled, between November 2020 and January 2022, adult HCWs employed in both public and private French establishments who were directly involved in the Covid‐19 outbreak.

Participants were recruited via hospital newsletters, emails and internet advertisements. To be included in the cohort, participants were required to meet the following criteria: aged 18 or older, fluent in French and employed as HCWs (including physicians, residents, nurses, nursing assistants, physiotherapists, paramedics and students). They had to be working in either a public or private hospital or in a Nursing Home for Dependent Elderly People, have provided care to patients with Covid‐19, possess health insurance coverage or entitlement, and demonstrate the capability to comply with the study's requirements.

### 2.4 Measures

All data were gathered through self‐reported online questionnaires. The variables included socio‐demographic characteristics (e.g., age, sex, marital status and profession), data on alcohol, cannabis, and psychotropic drug consumption, variables related to the experience of the Covid‐19 crisis, such as shortages of personal protective equipment (PPE) and deployment to a new unit. Previous research has demonstrated the significance of such contextual factors in influencing mental health outcomes among HCWs [14, 15, 16].

In our study, we evaluated three main psychological outcomes: PTSD symptoms, depressive symptoms and professional quality of life (ProQOL).

– PTSD was assessed using the PCL‐5 (PTSD Checklist), a 20‐item questionnaire aligning with DSM‐5 criteria for PTSD [20, 21]. In this study, a cut‐off score of ≥ 40 was used to indicate the likelihood of PTSD symptoms. We acknowledge that higher cut‐off scores imply more stringent inclusion criteria and a greater potential for false negatives.
– Depressive symptoms were assessed with the PHQ‐9 (Patient Health Questionnaire), a self‐administered questionnaire based on DSM‐5 criteria for depression. While the PHQ‐9 can aid in the diagnostic process, in our context, scores ranging from 0 to 27, with a cut‐off score of ≥ 15, were used to identify individuals experiencing moderately severe depressive symptoms [22].
– ProQOL was assessed with the ProQOL scale, a 30‐item self‐administered tool that evaluates the professional satisfaction of HCWs across two primary dimensions: compassion satisfaction and compassion fatigue (which includes burn‐out and secondary traumatic stress). Each dimension yields its own score and interpretation: a lower score ( ≤ 22) in the compassion satisfaction dimension indicates decreased professional fulfillment, a score ≥ 42 in the burn‐out dimension suggests a heightened risk for burn‐out and a score ≥ 42 in the secondary traumatic stress dimension may indicate symptoms of stress secondary to exposure to traumatic events experienced by others [23].

Other psychological assessments included the following measures:

– The 21‐item PTGI (Posttraumatic Growth Inventory) is a self‐report tool designed to assess positive outcomes following trauma. It measures four key factors: new life directions, relationships, personal capabilities and spiritual change [24]. Scores range from 0 to 105, with higher scores indicating greater positive growth after a traumatic event [25].
– The 28‐item CTQ (Childhood Trauma Questionnaire) is a self‐report tool used to assess traumatic experiences before age 18. It features five subscales: emotional abuse, physical abuse, sexual abuse, emotional neglect and physical neglect. Each item is rated on a 5‐point frequency scale, with scores reflecting the degree of abuse for each subscale [26, 27].
– The self‐report GAD‐7 (General Anxiety Disorder) scale is used to screen for GAD symptoms and assess their severity. Each item is scored from 0 to 3, with higher total scores indicating increased GAD symptom severity [28].
– The 28‐item self‐report Brief COPE (Coping Orientation to Problems Experienced Inventory) is used to evaluate individuals' coping strategies [29]. It assesses 14 distinct strategies [30, 31], with each item rated from 0 (not at all) to 3 (totally); higher scores indicate increased use of a specific coping mechanism. Here, we focused on four relevant coping strategies: social support, problem‐solving, avoidance and positive thinking (Baumstarck et al., 2017).
– The 8‐item self‐report P‐Scale (Positivity Scale) gauges an individual's tendency to view life and experiences with a positive orientation. Items are rated from 1 (strongly disagree) to 5 (strongly agree), with higher scores indicating greater positivity [32, 33].
– The 36‐item SF‐36 (Short‐Form questionnaire) evaluates perceived health across eight dimensions: physical functioning, social functioning, physical problems, emotional problems, mental health, vitality, pain and general health perception [34]. Higher scores indicate better health status [35, 36]. We focused solely on the dimensions of limitations in usual role activities due to emotional and physical problems.
– The PPP‐VAS (Physical and Psychological Pain—Visual Analogue Scale) assesses suicidal ideation and pain. This tool measures current, mean and worst PPP over the last 15 days. Similarly, current, mean and worst suicidal ideation, along with the history of suicidal attempts, are also assessed [37].

### 2.5 Statistics

The analysed sample comprised all participants in the cohort who completed at least one of the three main scales (i.e., PCL‐5, PHQ‐9 and/or ProQOL). Continuous data are presented as medians and interquartile ranges (1st to 3rd quartiles) or means and their standard deviations, as appropriate according to the data distribution, while categorical data are presented as counts and percentages. We selected a subset of variables to explore association with the three main psychological assessments. Our selection of variables to be included in this subset was guided by the variables identified as associated with psychological symptoms in the literature [38]. The subset included age, sex, student status, profession (physician or not), marital status, lack of PPE during Covid‐19 crisis, deployment to a new unit or activity during Covid‐19 crisis, P‐Scale and Brief COPE scale. Univariate analysis was conducted using Student's *t*‐tests, and bivariate Pearson correlation coefficients explored the association of the three main psychological scores with binary and continuous variables, respectively.

For each of the three main psychological scores, all variables with a *p* value < 0.20 in the univariate analysis were selected and included in a multiple linear regression to explore the factors influencing the score's values. For the Brief Cope, the four dimensions were included in the model as soon as one of them was associated with the dependent variable.

For each scale, we performed the imputation of missing items by the Personal Mean Score (PMS) (Leplege et al., 2001). PMS consists of imputing missing scores by the average of the item responses of the same dimension answered by the individual, if more than half of the items are filled in for this individual and this score. If more than half of the items were missing for a given score and individual, missing data were not imputed. The level of statistical significance was set to 0.05 (two‐tailed).

## 3 Results

**Figure 1:**
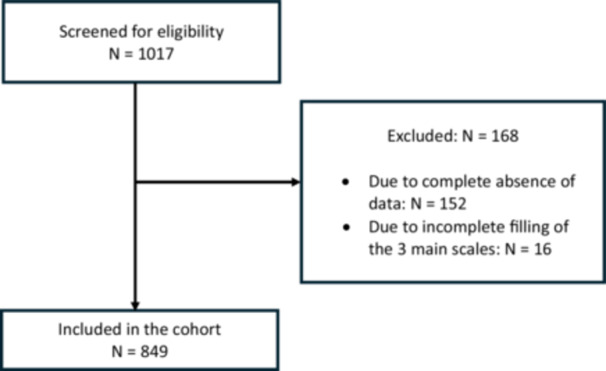
Flowchart.

Upon enrollment, 1,017 professionals were screened for eligibility. After excluding 168 individuals due to either complete absence of data (*n* = 152) or incomplete filling for all three main scales (*n* = 16), 849 HCWs were included in the cohort (Figure 1).

### 3.1 Socio‐Demographic Characteristics and Substance Consumption

Among the participants (mean age = 38.3 ± 10.2 years), 80.9% were women, and 27.7% were single. Most of the participants were nurses (62%) and 19.2% were physicians, with a median of 11.0 years of work experience [5.0–18.0] (Table 1). Regarding the use of psychotropic drugs, the most consumed class of drugs was anxiolytics, with 25% of all study participants reporting consumption. Among the subset of participants who reported using any psychoactive substance, a considerable proportion indicated an increase in consumption during the pandemic's peak—37.8% for alcohol, 22.7% for cannabis and 53.7% for psychotropic drugs.

**Table 1:** Socio‐demographic and psychological characteristics.

Characteristic	*n* (%)
Female (*n* = 844)	683 (80.9)
Single marital status (*n* = 839)	232 (27.7)
Profession (*n* = 847)	
Nurse, student, assistant	525 (62.0)
Physician, resident, student	180 (21.2)
Other	104 (12.3)
Physiotherapist	18 (2.1)
Radiology manipulator, student	9 (1.1)
Pharmacist, intern	6 (0.7)
Midwife	3 (0.0)
Odontologist	2 (0.2)
Physician (including resident) (*n* = 847)	163 (19.2)
Student (*n* = 847)	50 (5.9)
Current or past alcohol consumption (*n* = 744)	589 (79.2)
Frequency of alcohol consumption (*n* = 589)	
Once per month or less	113 (19.2)
2–4 times per month	262 (44.5)
2–3 times per week	147 (25.0)
More than 4 times per week	67 (11.4)
Increased alcohol use during the pandemic (*n* = 579)	219 (37.8)
Cannabis consumption (*n* = 680)	71 (10.4)
Cannabis consumption in the past month (*n* = 73)	
Less than once a month	61 (83.6)
Less than once a week	5 (6.8)
Several times a week	2 (2.7)
Everyday	5 (6.8)
Increase cannabis use during the pandemic (*n* = 75)	17 (22.7)
Increase in cannabis use in the past 6 months (*n* = 75)	14 (18.7)
Consumption of anxiolytics (*n* = 709)	177 (25.0)
Consumption of hypnotics (*n* = 699)	70 (10.0)
Consumption of antidepressants (*n* = 697)	58 (8.3)
Self‐medication with psychotropic drugs (*n* = 215)	124 (57.7)
Increase in psychotropic drug use during the pandemic (*n* = 203)	109 (53.7)

Abbreviations: CTQ, Childhood Trauma Questionnaire; GAD, Generalized Anxiety Disorder; PCL‐5, PTSD Checklist for DSM‐5; PHQ‐9, Patient Health Questionnaire; ProQOL, Professional Quality of Life; PTGI, Posttraumatic Growth Inventory; PTSD, post‐traumatic stress disorder; SF‐36, Short Form 36.

### 3.2 Covid‐19‐Related Characteristics

Nearly all participants had been in direct contact with patients infected with Covid‐19 (84.3%) or had worked in a Covid‐19 risk unit, such as the emergency unit, intensive care or a dedicated Covid‐19 unit (81.4%). Further, more than half participants (53.6%) reported a lack of PPE, a feeling of not being supported by the hospital, having doubts regarding the ability of the hospital to provide for personal or family needs in the event of a Covid‐19 infection or feeling a lack of information or communication from the hospital. Most participants had struggled with specific Covid‐19‐related challenging situations, such as experiencing uncertainty about the weeks ahead (89.4%), the suffering of families (84.2%) or experiencing patient deaths (69.2%).

The majority had relatives or colleagues affected by Covid‐19 (55.8% and 96.6%, respectively). Most participants reported pride in working during the pandemic (77.2%) (Table 2).

**Table 2:** Characteristics related to Covid‐19 experience.

Characteristics	*n* (%)
Direct contact with Covid patients (*n* = 809)	682 (84.3)
Lack of personal protective equipment (*n* = 806)	432 (53.6)
Lack of means to test for suspected Covid‐19 symptoms (*n* = 808)	260 (32.2)
Being deployed to a new unit/activity (*n* = 804)	376 (46.8)
Doubts about the ability of the hospital to provide for personal or family needs in the event of Covid‐19 infection (*n* = 803)	516 (64.3)
Work in a Covid risk unit (*n* = 808)	658 (81.4)
Feeling supported by the hospital (*n* = 803)	356 (44.3)
Feeling of lack of information/communication from the hospital (*n* = 806)	488 (60.5)
Satisfactory communication within the team (*n* = 804)	571 (71.0)
Have struggled through the following challenging situations	
Patient deaths (*n* = 804)	556 (69.2)
Absence of families (*n* = 800)	659 (82.4)
Families suffering (*n* = 795)	669 (84.2)
Away from your loved ones (*n* = 801)	549 (68.5)
Fear of contamination (*n* = 804)	488 (60.8)
Fear of making a technical mistake (*n* = 802)	451 (56.1)
Involvement in treatment limitation decisions for Covid‐19 (*n* = 797)	441 (55.3)
Pride in work during the pandemic (*n* = 797)	615 (77.2)
Affected by Covid‐19 (*n* = 803)	224 (27.9)
Hospitalised for Covid‐19 (*n* = 234)	13 (5.6)
Relatives affected by Covid‐19 (*n* = 799)	446 (55.8)
Relatives hospitalised because of Covid‐19 (*n* = 458)	125 (27.3)
Deceased relatives of Covid‐19 (*n* = 795)	69 (8.7)
Negative impact of the pandemic peak on personal and family relationships (*N* = 800)	510 (63.7)

### 3.3 PTSD, Depression and Burn‐Out

Prevalence of PTSD symptoms (PCL‐5 ≥ 40) was estimated at 17.7%, with a mean score of 24.2. Moderately severe depressive symptoms, defined by a score ≥ 15, were found in 14.0% of participants, and the mean score for the PHQ‐9 was 8.1 (Table 3). A minority of participants exhibited a high risk for burn‐out (0.9%), with mean scores for compassion satisfaction, burn‐out and secondary traumatic stress of 36.4, 26.6 and 24.1, respectively.

**Table 3:** Three main psychological evaluations.

	*N* = 849
PCL‐5	
Mean ± SD (*N* = 827)	24.2 ± 15.2
Score ≥ 40, *n* (%) (*N* = 827)	146 (17.7)
PHQ‐9	
Mean ± SD (*N* = 840)	8.1 ± 5.6
Score ≥ 15, *n* % (*N* = 840)	118 (14.0)
ProQOL	
Mean ± SD	
Satisfaction (*N* = 823)	36.4 ± 6.5
Burn‐out (*N* = 826)	26.6 ± 6.3
Secondary trauma (*N* = 828)	24.1 ± 7.2
Threshold score, *n* (%)	
Satisfaction severe ≤ 22 (*N* = 823)	18 (2.2)
Satisfaction moderate 23–41 (*N* = 823)	621 (75.5)
Burn‐out severe ≥ 42 (*N* = 826)	7 (0.8)
Burn‐out moderate 23–41 (*N* = 826)	597 (72.3)
Secondary trauma severe ≥ 42 (*N* = 828)	16 (1.9)
Secondary trauma moderate 23–41 (*N* = 828)	429 (51.8)

Abbreviations: PCL‐5, PTSD Checklist for DSM‐5; PHQ‐9, Patient Health Questionnaire; ProQOL, Professional Quality of Life; PTSD, post‐traumatic stress disorder; SD, standard deviation.

### 3.4 Other Psychological Outcomes

Notably, a mild to moderate level of anxiety was found among the participants (mean GAD score = 7.4), as well as a mean positivity score of 27.5. The health status assessment showed high scores in the emotional and physical roles subdimensions. Regarding suicidal ideation, 12% of the participants had a history of suicide attempt (Table 4).

**Table 4:** Other psychological evaluations.

	Mean ± SD
PTGI	
New direction for life (*n* = 778)	6.6 ± 5.0
Personal capabilities (*n* = 782)	7.6 ± 5.4
Spiritual change (*n* = 782)	1.1 ± 1.8
Relationships with others (*n* = 772)	7.0 ± 6.0
CTQ	
Emotional neglect (*n* = 778)	10.6 ± 4.6
Physical abuse (*n* = 778)	6.0 ± 2.6
Emotional abuse (*n* = 778)	8.6 ± 4.4
Physical neglect (*n* = 781)	6.5 ± 2.3
Sexual abuse (*n* = 777)	5.9 ± 2.4
GAD (*n* = 766)	7.4 ± 5.3
Brief Cope factors	
Social support (*n* = 758)	16.7 ± 4.6
Problem‐solving (*n* = 757)	8.7 ± 3.0
Avoidance (*n* = 758)	18.4 ± 4.4
Positive thinking (*n* = 757)	13.3 ± 3.4
Positivity Scale (*n* = 749)	27.5 ± 4.8
SF‐36	
Emotional (*n* = 742)	53.7 ± 40.3
Physical (*n* = 746)	55.8 ± 37.6

Abbreviations: CTQ, Childhood Trauma Questionnaire; GAD, Generalized Anxiety Disorder; PTGI, Posttraumatic Growth Inventory; SD, standard deviation; SF‐36, Short Form 36.

### 3.5 Univariate Analysis of the Three Main Psychometric Scores

The associations between selected variables and psychological outcomes—PTSD, depression and professional burn‐out—are detailed in Tables 5 and 6. Female gender, being a non‐physician, lacking PPE and redeployment to new units were each associated with significantly higher psychological distress across several outcomes. Specifically, higher PTSD symptoms (PCL‐5) were reported by females, non‐students, non‐physicians and those without PPE. Higher depression scores (PHQ‐9) were seen among females, non‐physicians, single individuals and those redeployed to new units. Burn‐out (ProQOL) was significantly more severe among females, non‐students, non‐physicians, individuals lacking PPE and those redeployed. Compassion satisfaction, in contrast, was higher among physicians and those with access to PPE, while secondary traumatic stress was higher in females, non‐physicians, those lacking PPE and redeployed individuals.

**Table 5:** Univariate analyses of the association between qualitative socio‐demographic characteristics and the three psychometric scores of interest during Covid‐19.

		*n*	Mean ± SD	*p* value
PCL‐5				
Sex	Male	158	19.6 ± 14.7	**< 0.001**
	Female	664	25.3 ± 15.2	
Student	Yes	49	19.4 ± 14.6	**0.022**
	No	776	24.5 ± 15.2	
Profession	Physician	159	19.6 ± 13.7	**< 0.001**
	Other	666	25.3 ± 15.4	
Marital status	Single	227	24.3 ± 15.1	0.918
	Couple	591	24.1 ± 15.3	
Lack of personal protective equipment	Yes	429	25.9 ± 15.6	**< 0.001**
	No	363	22.3 ± 14.7	
Deployment to a new unit/activity	Yes	371	25.3 ± 15.2	**0.072**
	No	419	23.3 ± 15.4	
PHQ‐9				
Sex	Male	159	6.6 ± 5.8	**< 0.001**
	Female	676	8.4 ± 5.5	
Student	Yes	49	7.8 ± 6.0	0.729
	No	789	8.1 ± 5.6	
Profession	Physician	157	6.6 ± 5.5	**< 0.001**
	Other	681	8.4 ± 5.6	
Marital status	Single	230	8.8 ± 5.8	**0**.**020**
	Couple	600	7.8 ± 5.5	
Lack of personal protective equipment	Yes	432	8.5 ± 5.6	**0.055**
	No	374	7.7 ± 5.6	
Deployment to a new unit/activity	Yes	376	8.7 ± 5.6	**0.011**
	No	428	7.6 ± 5.5	
ProQOL, burn‐out				
Sex	Male	157	25.1 ± 6.6	**< 0.001**
	Female	664	27.0 ± 6.2	
Student	Yes	47	24.8 ± 0.0	**0.039**
	No	777	26.7 ± 6.3	
Profession	Physician	155	25.4 ± 6.7	**0.009**
	Other	228	26.7 ± 6.2	
Marital status	Single	588	26.6 ± 6.4	0.798
	Couple	600	7.8 ± 5.5	
Lack of personal protective equipment	Yes	431	27.5 ± 6.3	**< 0.001**
	No	372	25.6 ± 6.3	
Deployment to a new unit/activity	Yes	373	27.2 ± 6.3	**0.023**
	No	428	26.2 ± 6.4	
ProQOL, satisfaction				
Sex	Male	155	37.0 ± 7.4	0.178
	Female	663	36.3 ± 6.3	
Student	Yes	47	38.1 ± 7.4	**0.063**
	No	774	36.3 ± 6.4	
Profession	Physician	155	37.8 ± 6.4	**0.003**
	Other	666	36.1 ± 6.5	
Marital status	Single	228	35.8 ± 6.6	**0.102**
	Couple	585	36.7 ± 6.5	
Lack of personal protective equipment	Yes	431	35.9 ± 6.5	**0.022**
	No	371	37.0 ± 6.5	
Deployment to a new unit/activity	Yes	373	36.1 ± 6.4	0.291
	No	427	36.6 ± 6.6	
ProQOL, secondary trauma				
Sex	Male	158	21.9 ± 7.0	**< 0.001**
	Female	665	24.6 ± 7.1	
Student	Yes	47	23.0 ± 6.9	0.277
	No	779	24.1 ± 7.2	
Profession	Physician	155	22.9 ± 6.6	**0.024**
	Other	671	24.3 ± 7.3	
Marital status	Single	228	23.7 ± 7.1	0.456
	Couple	590	24.2 ± 7.2	
Lack of personal protective equipment	Yes	431	24.9 ± 7.3	**< 0.001**
	No	372	23.1 ± 7.0	
Deployment to a new unit/activity	Yes	373	24.6 ± 7.3	**0.034**
	No	428	23.6 ± 7.1	

*Note: p* values of Student's *t*‐test ≤ 0.05 are in bold.

Abbreviations: PCL‐5, PTSD Checklist for DSM‐5; PHQ‐9, Patient Health Questionnaire; ProQOL, Professional Quality of Life; PTSD, post‐traumatic stress disorder; SD, standard deviation.

**Table 6:** Correlation between quantitative characteristics and the three psychometric scores of interests during Covid‐19.

	*n*	Pearson Rhô	95% CI of Rhô	*p* value
PCL‐5				
Brief Cope				
Social support	742	0.14	[0.07; 0.21]	**< 0.001**
Problem‐solving	741	0.07	[0; 0.15]	**0.04**
Avoidance	742	0.55	[0.49; 0.60]	**< 0.001**
Positive thinking	741	−0.32	[−0.38; −0.25]	**< 0.001**
Positivity Scale	733	−0.38	[−0.44; −0.32]	**< 0.001**
PHQ‐9				
Brief‐Cope				
Social support	756	0.11	[0.04; 0.18]	**0.003**
Problem‐solving	755	0.02	[−0.05; 0.09]	0.61
Avoidance	756	0.50	[0.45; 0.55]	**< 0.001**
Positive thinking	755	−0.26	[−0.33; −0.20]	**< 0.001**
Positivity Scale	747	−0.45	[−0.51; −0.39]	**< 0.001**
ProQOL, burn‐out				
Brief‐Cope				
Social support	753	0.02	[−0.06; 0.09]	0.66
Problem‐solving	752	−0.06	[−0.13; 0.01]	0.11
Avoidance	753	0.45	[0.40; 0.51]	**< 0.001**
Positive thinking	752	−0.37	[−0.43; −0.31]	**< 0.001**
Positivity Scale	746	−0.50	[−0.55; −0.44]	**< 0.001**
ProQOL, satisfaction				
Brief Cope				
Social support	752	0.11	[0.04; 0.18]	**0.002**
Problem‐solving	751	0.21	[0.15; 0.28]	**< 0.001**
Avoidance	752	−0.25	[−0.32; −0.18]	**< 0.001**
Positive thinking	751	0.37	[0.31; 0.43]	**< 0.001**
Positivity Scale	745	0.52	[0.47; 0.57]	**< 0.001**
ProQOL secondary trauma				
Brief‐Cope				
Social support	753	0.18	[0.11; 0.25]	**< 0.001**
Problem‐solving	752	0.09	[0.02; 0.16]	**0.017**
Avoidance	753	0.51	[0.45; 0.56]	**< 0.001**
Positive thinking	752	−0.27	[−0.34; −0.20]	**< 0.001**
Positivity Scale	746	−0.33	[−0.39; −0.26]	**< 0.001**

*Note: p* values ≤ 0.04 are in bold.

Abbreviations: PCL‐5, PTSD Checklist for DSM‐5; PHQ‐9, Patient Health Questionnaire; ProQOL, Professional Quality of Life; PTSD, post‐traumatic stress disorder.

Correlation between psychological variables (such as coping strategies and positivity) and the three main outcomes is presented in Table 6. Avoidant coping strategies showed consistent moderate to strong positive correlations with higher symptoms across PTSD, depression, burn‐out and secondary trauma. Conversely, positive thinking strategies and higher scores on the P‐Scale were moderately associated with lower psychological distress and higher professional satisfaction. These findings suggest that cognitive and emotional coping resources may serve as protective factors, while avoidant strategies may exacerbate distress.

Overall, the most consistent and clinically relevant associations involved gender (female), occupational role (non‐physician), inadequate PPE and redeployment—highlighting these as key risk markers for psychological distress in HCWs during the pandemic. The associations ranged from small to moderate in magnitude, suggesting meaningful but not overwhelming effects.

### 3.6 Multivariate Analysis of the Three Main Psychometric Scores

Higher levels of PTSD symptoms were significantly associated with physician profession, lack of PPE, problem‐solving and avoidance coping strategies, and lower levels of positive thinking and positivity (Table 7).

**Table 7:** Multivariate regression of the three main psychological scores according to socio‐demographic and Covid factors, brief‐Cope and Positivity Scale.

	Beta	95% CI	*p* value
PCL‐5			
Intercept	17.26	[9.42; 25.10]	**< 0.001**
Female (Ref = Male)	1.04	[−1.25; 3.34]	0.373
Student (Ref = Other)	−1.79	[−5.37; 1.79]	0.326
Physician (Ref = Non‐physician profession)	−3.50	[−5.78; −1.21]	**0.003**
Lack of personal protective equipment (Ref = No)	2.24	[0.51; 3.97]	**0.011**
Deployment to a new unit/activity (Ref = No)	0.18	[−1.57; 1.93]	0.844
Brief Cope			
Social support	0.12	[−0.09; 0.32]	0.275
Problem‐solving	0.80	[0.48; 1.13]	**< 0.001**
Avoidance	1.44	[1.22; 1.66]	**< 0.001**
Positive thinking	−0.93	[−1.23; −0.64]	**< 0.001**
Positivity Scale	−0.64	[−0.85; −0.42]	**< 0.001**
PHQ‐9			
Intercept	10.06	[7.03; 13.08]	**< 0.001**
Female (Ref = Male)	0.20	[−0.68; 1.07]	0.659
Physician (Ref = Non‐physician profession)	−1.07	[−1.93; −0.21]	**0.015**
Marital status: In a couple (Ref = Single)	−0.45	[−1.18; 0.28]	0.230
Lack of personal protective equipment (Ref = No)	0.17	[−0.48; 0.82]	0.611
Deployment to a new unit/activity (Ref = No)	0.56	[−0.10; 1.22]	0.094
Brief Cope			
Social support	0.05	[−0.03; 0.13]	0.242
Problem‐solving	0.17	[0.05; 0.29]	**0.007**
Avoidance	0.44	[0.36; 0.52]	**< 0.001**
Positive thinking	−0.15	[−0.26; ‐0.04]	**0.010**
Positivity Scale	−0.38	[−0.46; ‐0.30]	**< 0.001**
ProQOL, burn‐out			
Intercept	33.27	[29.99; 36.54]	**< 0.001**
Female (Ref = Male)	0.14	[−0.83; 1.10]	0.781
Student (Ref = Other)	−1.13	[−2.64; 0.37]	0.139
Physician (Ref = Non‐physician profession)	−0.25	[−1.21; 0.70]	0.602
Lack of personal protective equipment (Ref = No)	1.21	[0.50; 1.93]	**< 0.001**
Deployment to a new unit/activity	0.27	[−0.46; 1.00]	0.472
Brief Cope			
Social support	−0.02	[−0.10; 0.07]	0.690
Problem‐solving	0.13	[0.00; 0.27]	0.052
Avoidance	0.45	[0.36; 0.54]	**< 0.001**
Positive thinking	−0.36	[−0.49; ‐0.24]	**< 0.001**
Positivity Scale	−0.43	[−0.52; ‐0.34]	**< 0.001**
ProQOL, satisfaction			
Intercept	17.71	[14.09; 21.33]	**< 0.001**
Female (Ref = Male)	1.03	[−0.02; 2.07]	0.055
Student (Ref = Other)	1.21	[−0.47; 2.89]	0.158
Physician (Ref = non‐physician profession)	0.50	[−0.53; 1.54]	0.339
Marital status: In a couple (Ref = Single)	0.57	[−0.33; 1.47]	0.216
Lack of personal protective equipment (Ref = No)	−0.46	[−1.24; 0.32]	0.243
Brief Cope			
Social support	0.05	[−0.04; 0.14]	0.296
Problem‐solving	0.11	[−0.04; 0.25]	0.150
Avoidance	−0.16	[−0.26; ‐0.06]	**0.001**
Positive thinking	0.30	[0.17; 0.44]	**< 0.001**
Positivity Scale	0.54	[0.44; 0.63]	**< 0.001**
ProQOL secondary trauma			
Intercept	18.34	[14.41; 22.27]	**< 0.001**
Female vs Male	0.93	[−0.22; 2.08]	0.112
Physician versus non‐physician profession	−0.35	[−1.49; 0.78]	0.541
Lack of personal protective equipment (Ref = No)	1.09	[0.23; 1.95]	**0.013**
Deployment to a new unit/activity	0.27	[−0.61; 1.14]	0.549
Brief Cope			
Social support	0.12	[0.02; 0.23]	**0.019**
Problem‐solving	0.31	[0.15; 0.47]	**< 0.001**
Avoidance	0.64	[0.53; 0.75]	**< 0.001**
Positive thinking	−0.38	[−0.53; ‐0.23]	**< 0.001**
Positivity Scale	−0.26	[−0.36; ‐0.15]	**< 0.001**

*Note: p* values ≤ 0.02 are in bold.

Abbreviations: PCL‐5, PTSD Checklist for DSM‐5; PHQ‐9, Patient Health Questionnaire; ProQOL, Professional Quality of Life; PTSD, post‐traumatic stress disorder.

Higher levels of depression symptoms were significantly associated with non‐physician professions, problem‐solving and avoidance coping strategies, and lower levels of positive thinking and positivity.

Higher ProQOL burn‐out scores were significantly associated with a lack of PPE, avoidance coping, and lower levels of positive thinking and positivity, while higher ProQOL compassion satisfaction scores are associated with lower avoidance coping, higher positive thinking, higher positivity, and higher ProQOL secondary trauma scores are associated with lack of PPE, social support, problem‐solving, avoidance coping and lower levels of positive thinking and positivity.

## 4 Discussion

This study presents the findings of the baseline data from the HARD study, which was conducted to estimate the prevalence of symptoms of PTSD, depression and professional burn‐out among HCWs during the Covid‐19 outbreak and to explore the variables associated with these psychological outcomes in this population [19].

A total of 849 HCWs were included in our study, primarily single women and nurses, with a notable proportion reporting the use of psychotropic substances. It is important to highlight that our sample was not intended to be representative of all French HCWs but rather consisted of individuals who responded to a call for participation in a psychological intervention during the Covid‐19 pandemic—potentially those already experiencing psychological distress. This may partly explain the high reported rates of psychotropic substance use. However, national data also indicate an increase in substance use among French HCWs during the pandemic. For example, in a large national survey (Rolland et al. 2025), psychotropic treatment use increased from 7.3% to 18% between 2020 and 2021, and psychoactive substance use rose from 13% to 21%. Another study reported that 36.4% of French physicians were hazardous drinkers [39]. In our study, nurses appeared particularly vulnerable, with a higher prevalence of burn‐out compared to other HCWs, a finding consistent with prior literature linking this to high workloads and sustained exposure to a life‐threatening disease (Hu et al. 2020; Jang et al. 2021).

In our study, 17% of participants had a PCL‐5 score indicating the presence of probable PTSD, 14% showed moderately severe depression, and 73% were at moderate risk for burn‐out. These findings align with recent reviews, showing a high prevalence of these three main psychological outcomes among HCWs pre‐ and post‐pandemic (West et al. 2016; Rotenstein et al. 2018 [12]; Salari et al. 2020; Duarte et al. 2022). We also reported additional psychological outcomes, indicating mild to moderate symptoms of anxiety among participants, as well as a higher proportion of respondents having attempted suicide in their lifetime compared to the general population. Previous studies have shown that suicidal behaviour is more frequent among HCWs, with a risk of suicide attempt 3–5 times higher than the general population (Kalmoe 2019; Dutheil et al. 2019), and even more so during the pandemic (García‐Iglesias et al. 2022; Dubé et al. 2021).

The aim of our study was also to explore the factors associated with the major psychological outcomes we assessed. Numerous works in the literature have highlighted several risk factors for PTSD, burn‐out and depression among HCWs in the context of a pandemic. Lai et al. showed that working as ‘a frontline HCWs with direct engagement of patients with COVID‐19’ was an independent risk factor for these psychological symptoms. Additionally, a high workload, respiratory or digestive symptoms, a negative coping style, and undergoing specific tests related to Covid‐19 were shown to be independent risk factors for anxiety and depression among HCWs (Chen et al. 2021). Other risk factors included a lack of protective measures, professional title and gender, with women being more at risk of experiencing stress and anxiety (Xiao et al. 2020).

Our results are consistent with these prior data. We found that being a woman, not being a physician and lacking protective equipment were associated with a higher risk for PTSD, burn‐out and depression. Further, we found that being a woman and not being a physician were independently associated with a higher risk for these negative psychological outcomes. Specifically, our finding that women are more vulnerable to these negative outcomes is supported by research from Safiye et al. [40, 41], which also highlighted the role of gender, inadequate rest, frontline Covid‐19 work and depersonalisation in burn‐out and mental health among HCWs during the Covid‐19 pandemic. The findings of Safiye et al. [41] further indicate that older age, religiosity, larger households and higher socio‐economic status were protective factors against burn‐out among Serbian HCWs. Similarly, Safiye et al. [40] emphasise the importance of resilience and hypermentalising as significant negative predictors of depression, anxiety and stress, while hypomentalising emerged as a significant positive predictor for these outcomes. These studies suggest that a comprehensive understanding of the impact of a pandemic on HCWs' well‐being necessitates consideration of a complex interplay of factors, including gender, working conditions and mental health support, to mitigate burn‐out and enhance well‐being during medical crises. The consistency of these findings strengthens the evidence base for targeted interventions.

Our findings of a high positivity level being a protective factor against these psychological outcomes are also in line with previous data suggesting that satisfaction with life was associated with lower levels of burn‐out, depression, stress and anxiety (Duarte et al. 2022; Uchmanovicz et al. 2019).

This study reinforces the need for multilevel prevention strategies to protect the mental health of HCWs. It has been shown that poor mental health among HCWs is associated with poor patient safety outcomes [42]. These measures can be categorised into primary prevention (such as ensuring adequate protective measures and reasonable workloads), secondary prevention (including systematic screening of at‐risk HCWs for early detection of mental health difficulties) and tertiary prevention (offering timely access to evidence‐based treatments). The variables highlighted in this study are targets for preventing poor psychological outcomes, such as ensuring a sufficient supply of PPE during a pandemic, and providing more medical support.

The high prevalence of these psychological outcomes suggests that repeated screening programmes should be conducted, especially in at‐risk populations like women and non‐medical professions. Though certain treatment strategies have shown their efficacy, more interventions are being currently evaluated to expand the therapeutic arsenal, including in EMDR in our randomised trial embedded in this cohort study [19].

The strengths of our study include its large sample size, although it is less than the expected one, and the high response rate, with data collected from several healthcare centres, providing a representative sample of HCWs. However, our study has some limitations. It is crucial to interpret our findings within the context of our recruitment strategy and study design. Online recruitment through social media advertising may have led to selection bias, with HCWs suffering from psychological symptoms being more prone to participate. Such bias could have led to overestimating the prevalence of PTSD, depression and burn‐out. Nonetheless, the results we found are in line with previous data, suggesting a moderate effect of this bias. Data collection is based on a self‐administered questionnaire, and future research should focus on clinician‐administered measures to account for reporting bias and to confirm formal diagnoses of PTSD, depression and burn‐out.

## 5 Conclusion

Although our sample may over‐represent HCWs with psychological difficulties—given the voluntary nature of participation in a study advertised as a psychological intervention—our findings remain consistent with international data and underscore a critical reality: symptoms of PTSD, depression and burn‐out were prevalent among French HCWs during the Covid‐19 pandemic. Women and non‐physician professionals, such as nurses, appear particularly vulnerable. These results highlight the urgent need for targeted, multilevel interventions. At the governmental level, this includes sustained investment in mental health infrastructure within healthcare systems, dedicated psychological support programmes for HCWs, and stronger occupational health protections during crises. At the institutional level, hospitals and health centres should implement systematic mental health screening, improve access to psychological services, and support peer‐support initiatives and team‐based debriefings. At the team level, training for supervisors to recognise early signs of distress, the promotion of supportive work environments, and efforts to reduce stigma around seeking help are essential. Such initiatives should be tailored to those most at risk and integrated into pandemic preparedness plans to protect the healthcare workforce in future crises.

## Author Contributions

**Wissam El‐Hage:** conceptualization, methodology, supervision, writing – review and editing, funding acquisition. **Alexandre Lemé:** writing – original draft, writing – review and editing. **Myriam Blanchin:** formal analysis, software, resources, data curation, writing – review and editing. **Eric Bui:** investigation, writing – review and editing. **Hala Kerbage:** investigation, writing – review and editing. **Sarah Ibouhsissen:** formal analysis, software, resources, data curation, writing – review and editing. **Aude Allemang‐Trivalle:** formal analysis, software, resources, data curation, writing – review and editing. **Valérie Gissot:** methodology, investigation, project administration, writing – review and editing. **Bénédicte Gohier:** investigation, writing – review and editing. **Coraline Hingray:** investigation, writing – review and editing. **Philippe Birmes:** investigation, writing – review and editing. **Eric Fakra:** investigation, writing – review and editing. **Nathalie Prieto:** investigation, writing – review and editing. **Cédric Lemogne:** investigation, writing – review and editing. **Marie‐Odile Krebs:** investigation, writing – review and editing. **Bruno Aouizerate:** investigation, writing – review and editing. **Isabelle Jalenques:** investigation, writing – review and editing. **Pierre Vidailhet:** investigation, writing – review and editing. **Anne Sauvaget:** investigation, writing – review and editing. **Agnès Caille:** methodology, data curation, formal analysis, writing – review and editing.

## Disclosure

The funder has no role in study design; in the collection, analysis and interpretation of data; in writing of the report; and in the decision to submit the paper for publication.

## Conflicts of Interest

The authors declare no conflicts of interest.

## Patient or Public Contribution

This study presents the baseline findings of the larger HARD (HealthcAre woRkers coviD‐19) study. While this specific analysis focuses solely on initial cohort data, the overarching HARD study was significantly shaped by active Healthcare workers' (HCWs) involvement (PPI). As the primary focus of our research, HCWs' perspectives were integral to every stage of the study's design and execution. For instance, their crucial input during pre‐study discussions led us to integrate personalised feedback of psychological scores to empower participants with their own well‐being information. Furthermore, HCWs strongly advocated for and influenced the design of the psychological intervention, ensuring the trial offered tangible support to those with significant symptoms. Their invaluable feedback also guided our recruitment strategies, emphasising accessible communication channels, and streamlined our data collection methods for conciseness. Crucially, HCWs highlighted the necessity of a telehealth‐based approach for data collection and identifying a psychotherapist near home for the intervention delivery, a foresight that proved vital for the study's feasibility and accessibility during the pandemic and subsequent lockdowns. This deep engagement ensured the HARD study remained highly relevant to HCWs' experiences and directly addressed their specific needs and concerns.

## Data Availability

The data that support the findings in this study are available from the corresponding author upon reasonable request.
